# Model-Based Correction of Temperature-Dependent Measurement Errors in Frequency Domain Electromagnetic Induction (FDEMI) Systems

**DOI:** 10.3390/s22103882

**Published:** 2022-05-20

**Authors:** Martial Tazifor, Egon Zimmermann, Johan Alexander Huisman, Markus Dick, Achim Mester, Stefan Van Waasen

**Affiliations:** 1 Central Institute of Engineering, Electronics and Analytics (ZEA-2), Forschungszentrum Jülich GmbH, 52428 Jülich, Germany; e.zimmermann@fz-juelich.de (E.Z.); m.dick@fz-juelich.de (M.D.); a.mester@fz-juelich.de (A.M.); s.van.waasen@fz-juelich.de (S.V.W.); 2 Institute of Bio- and Geosciences, Agrosphere (IBG-3), Forschungszentrum Jülich GmbH, 52428 Jülich, Germany; s.huisman@fz-juelich.de

**Keywords:** electromagnetic induction (EMI), frequency domain electromagnetic induction (FDEMI) systems, apparent electrical conductivity (ECa), low-pass filter (LPF), data acquisition unit (DAQ), root mean square error (RMSE), drift correction

## Abstract

Data measured using electromagnetic induction (EMI) systems are known to be susceptible to measurement influences associated with time-varying external ambient factors. Temperature variation is one of the most prominent factors causing drift in EMI data, leading to non-reproducible measurement results. Typical approaches to mitigate drift effects in EMI instruments rely on a temperature drift calibration, where the instrument is heated up to specific temperatures in a controlled environment and the observed drift is determined to derive a static thermal apparent electrical conductivity (ECa) drift correction. In this study, a novel correction method is presented that models the dynamic characteristics of drift using a low-pass filter (LPF) and uses it for correction. The method is developed and tested using a customized EMI device with an intercoil spacing of 1.2 m, optimized for low drift and equipped with ten temperature sensors that simultaneously measure the internal ambient temperature across the device. The device is used to perform outdoor calibration measurements over a period of 16 days for a wide range of temperatures. The measured temperature-dependent ECa drift of the system without corrections is approximately 2.27 mSm^−1^K^−1^, with a standard deviation (std) of only 30 μSm^−1^K^−1^ for a temperature variation of around 30 K. The use of the novel correction method reduces the overall root mean square error (RMSE) for all datasets from 15.7 mSm^−1^ to a value of only 0.48 mSm^−1^. In comparison, a method using a purely static characterization of drift could only reduce the error to an RMSE of 1.97 mSm^−1^. The results show that modeling the dynamic thermal characteristics of the drift helps to improve the accuracy by a factor of four compared to a purely static characterization. It is concluded that the modeling of the dynamic thermal characteristics of EMI systems is relevant for improved drift correction.

## 1. Introduction

Electromagnetic induction (EMI) measurements allow a fast non-contact measurement of the soil apparent electrical conductivity (ECa), which is directly related to the bulk soil electrical conductivity and thus to soil properties such as clay content, salinity and water content. EMI measurements have been used for soil mapping in precision agriculture in a wide range of studies [1,2,3,4]. For instance, Cameron et al. [5], Visconti and Miguel de Paz [6] and Corwin and Rhoades [7] found that EMI allowed the fast mapping of the soil to obtain a clear delineation of field-scale salinity profiles. A range of EMI studies have also targeted hydrological properties to relate EMI measurements to soil water content and ground water dynamics [8,9,10]. For example, Kachanoski et al. [11] used EMI measurements to show that bulk soil electrical conductivity accounts for up to 96% of the spatial variation in soil water content.

In frequency domain EMI systems, a transmitter coil (Tx) is energized with an alternating current to produce a time-varying primary electromagnetic field that diffuses into the ground. As described by Faraday’s law of induction, this will induce eddy currents in the conductive subsurface that are proportional to the conductivity of the soil but shifted in phase with respect to the primary current from the Tx. These eddy currents generate a secondary magnetic field, whose strength depends on the subsurface electrical conductivity, the intercoil spacing and orientation and the operating frequency. The superposition of the primary and secondary magnetic fields is sensed at the receiver coil (Rx). The ratio of secondary to primary magnetic field has an in phase (real component) and a quadrature phase (imaginary component) and it can be shown that the quadrature phase is proportional to the electrical conductivity of the conductive subsurface [12].

**Figure 1:**
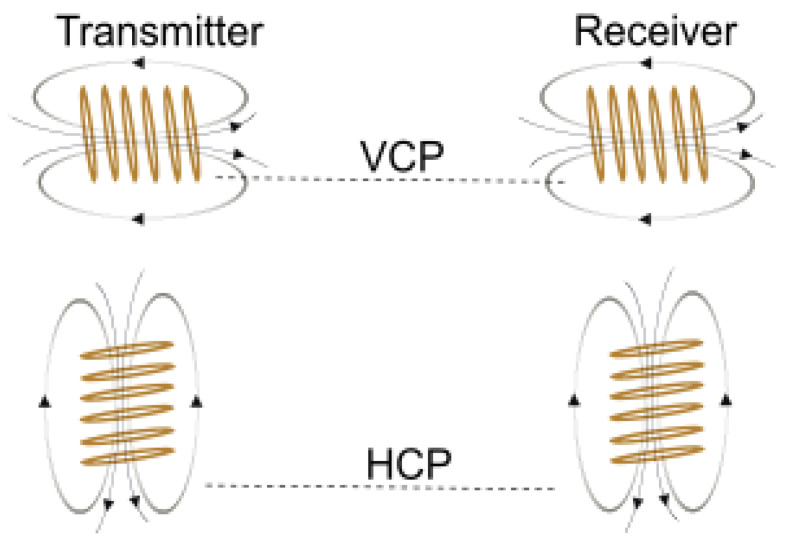
The vertical coplanar and horizontal coplanar coil configurations. The coils in VCP configuration have a dipole (arrow) horizontal to the plane of the soil. The coils in HCP configuration have a dipole (arrow) perpendicular to the plane of the surface.

EMI measurements can be obtained with different coil configurations, which results in differences in the induced eddy currents and the associated secondary magnetic field. The most popular coil configurations used for EMI measurements are the vertical coplanar (VCP) and horizontal coplanar (HCP) configurations (Figure 1). The HCP configuration has effectively twice the exploration depth of the VCP configuration. The sensitivity of the VCP configuration is highest at the surface and decreases with depth, while the sensitivity of the HCP configuration peaks at a depth of 0.4 times the intercoil separation [12,13]. Multi-coil EMI instruments consisting of a single Tx coil and multiple Rx coils are increasingly being used [14,15,16]. Additionally, multi-frequency EMI instruments operating at different frequencies are also widely used [15,17,18]. Both multi-coil and multi-frequency EMI systems offer the advantage that sensing over different depth ranges can be done, with the accuracy of measured ECa depending, on the one hand, on coil separation and configuration [18,19] and, on the other hand, on frequency [17,18].

The accuracy of EMI measurements has been a topic of concern in many studies as the measurements are sensitive to external ambient conditions. Temperature generally has the greatest effect on the drift of EMI instruments. Due to temperature dependencies, Abdu et al. [20] recommend mapping on cooler days or protecting the instruments from direct sunlight. Sudduth et al. [21] performed investigations with the EM38 instrument by varying ambient temperatures from 23 °C to 35 °C over 8 h and found that the measured ECa rose from 32.2 mSm^−1^ to 42.3 mSm^−1^. Huang et al. [22] showed that changes in ECa measured using the DUALEM–41S and DUALEM–21S instruments were also affected by ambient temperature variations. Partial shading of EMI instruments causes even more extreme and difficult-to-correct drift effects in the measurement data. For instance, Robinson et al. [23] performed experiments to investigate the stability of the temperature calibration of the EM38, and found that differential ambient heating by direct exposure to sunlight is one of the main reasons for drifts in measured EMI data. Clearly, there is therefore a need to compensate EMI data for signal drift.

Some studies have attempted to correct temperature-dependent drifts in EMI systems by analyzing the internal compensation circuits of commercial EMI instruments [20,21] and by using optimization, but they were typically not able to satisfactorily mitigate the effect of temperature on measured EMI data. Robinson et al. [23] claimed that the used internal compensation circuit of the EM38 instrument was unable to fully compensate for instrument heating for temperatures > 40 °C. They concluded that the drifts are a combination of instrument characteristics, such as the circuit design, and the performance of the instrument components under heating. Mester et al. [24] analyzed the effects of temperature drift on a custom EMI instrument. They corrected the significant thermal drifts of the receiver coils by measuring the temperature-dependent coil impedances. However, a complete correction of all drifts caused by various system components was not performed.

One method to improve the accuracy of EMI systems is drift calibration of the full EMI instrument using a look-up table. This is typically done by heating the measurement system up in an environment with controllable temperature. For system calibration with a range of steady-state temperatures, it is important to wait the required delay time after each temperature change until the EMI device returns to a stable state. A look-up table that relates temperature and drift can thus be established. Robinson et al. [23], Abdu et al. [20] and Hanssens et al. [25] performed such measurements and showed that it was possible to determine the relationship between signal drift and ambient temperature to obtain corrected EMI measurements. However, this method has at least two disadvantages. First, the derivation of such look-up tables is cumbersome and not only requires a suitable temperature-controlled room, but also requires that, for each calibration step, the delay time for attaining steady-state temperatures must be observed. Standard rooms in laboratories cannot be used for this purpose due to the influence of metals and electromagnetic interference. To avoid such interferences, outdoor measurements with thermal isolation boxes and cooling systems are an alternative for calibration measurements [26]. However, it is very difficult to achieve a homogeneous temperature with an accuracy better than 1 K in an outdoor environment. Second, the correction with look-up tables is only effective for stable temperature distributions or slow temperature variations. Drifts caused by abrupt changes in ambient temperature are more difficult to correct than drifts due to slow ambient temperature variations. This was demonstrated by Tan et al. [26], who showed that there is a delayed response between instrument temperature and measured ECa. Huang et al. [22] also observed hysteresis effects while studying temperature-dependent drifts. These studies thus suggest that it is currently not possible to efficiently correct drifts caused by rapid changes in ambient temperatures using look-up tables only.

The aim of this paper is to develop a novel drift error correction procedure that mitigates measurement errors associated with rapid ambient temperature fluctuations for typical EMI measurements on sunny cloud-free days with internal temperatures of up to 50 °C. The novel procedure involves a numerical drift model to reconstruct the temperature-dependent dynamic drift characteristics of the EMI instruments and will help to better understand the effects of temperature variation on EMI instruments. In addition, it provides a simple strategy to calibrate an EMI system using outdoor measurements.

In the following sections, the EMI system with integrated temperature sensors used for the investigation is presented first. Next, the drift model for error correction is introduced. In addition, the calibration measurement setup and the corresponding optimization methods to determine the model calibration parameters are presented. Finally, the main results are presented and discussed and the conclusions are provided.

## 2. Materials and Methods

### 2.1. EMI Measurement System with Temperature Sensors

**Figure 2:**
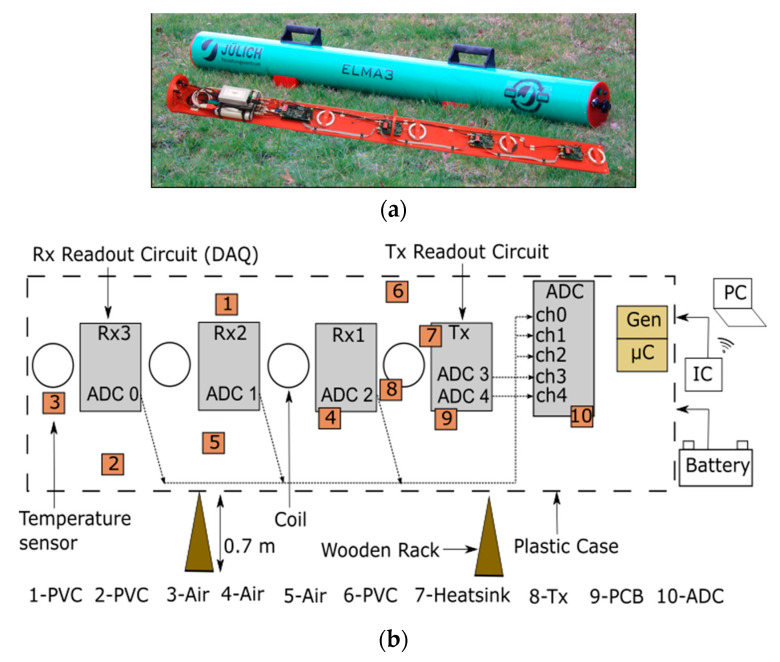
(**a**) Photo of the electromagnetic induction (EMI) instrument; (**b**) simplified representation of the EMI measurement setup. A polyvinyl chloride (PVC) pipe carries a transmitter (Tx) coil and three receiver (Rx) coils together with their readout circuits. An analog to digital converter (ADC), a generator unit (Gen), a microcontroller (μC) and an integrated computer (IC) all constitute the data acquisition unit (DAQ). The entire system is powered by an external battery and controlled remotely with the help of WLAN and a personal computer (PC). Sensor 9 measures the temperature of the printed circuit board (PCB); sensors 3, 4 and 5 monitor the air temperature inside the PVC tube; sensors 1, 2 and 6 measure the PVC temperature; sensor 8 measures the temperature at the Tx coil; sensor 7 measures the temperature of the heat sink, and sensor 10 measures the temperature of the ADC casing.

We use a customized EMI measurement system designed by Mester et al. [24] and described by Tan et al. [26]. It consists of one Tx and three Rx coils, a generator unit (Gen) to power the transmitting coil, a microcontroller (μC) for measuring temperatures and for hardware configuration, an integrated computer (IC) and an analog to digital converter (ADC) (Figure 2). The intercoil spacings are 0.4 m, 0.8 m and 1.2 m and the operating frequency is 10 kHz. This study is focused on the intercoil spacing of 1.2 m. The measurement instrument is powered by an external 12 V DC power supply (battery). The measurement data from the coils are collected and processed by means of an ADC. The ADC is a 24-bit sigma-delta ADC (National Instruments USB-4432) with a rated input voltage range of ±10 V and a resolution of 0.1 μV/√Hz. Each Rx coil has a readout circuit to amplify and transmit the measured signal to the ADC. The measurement system is operated by means of customized MATLAB software executed in the IC. The measurement program is controlled remotely over wireless LAN from an external notebook (PC). After the measurements, the data are transferred from the IC to the PC. The circuit design has been optimized for low drift effects, but without any active compensation circuits.

Ten temperature sensors were distributed within the EMI device to determine the temperature distribution and the temperature of specific components. The temperature sensors were placed at different locations, ranging from the generator and polyvinyl chloride (PVC) pipe on one end of the device to the Rx coils and readout circuits on the other end. The temperature measurements were made with ten digital thermometers (Dallas Semiconductor DS18S20), which have a measurement range from –55 °C to 125 °C with ± 0.5 °C accuracy and a 9-bit resolution.

**Figure 3:**
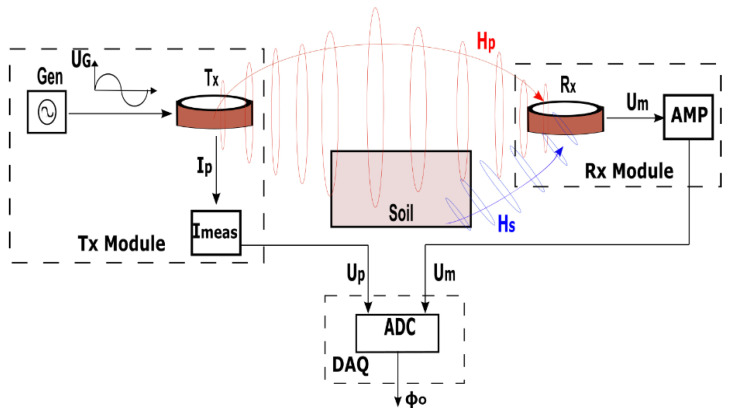
Signal flow diagram for the EMI measurement system for a single Tx–Rx arrangement.

Details on the EMI measurement system are shown on the signal flow diagram with a single Tx–Rx arrangement in Figure 3. The generator (Gen) supplies a sinusoidally varying voltage U_G_ at a specific frequency to the Tx coil. This produces a sinusoidal primary current I_p_ in the Tx coil and subsequently a primary magnetic field H_p_. The current I_p_ is determined through a current measurement circuit I_meas_, which measures the voltage drop U_p_ over a shunt resistor connected serially to the Tx coil. Ideally, the phase of H_p_ and U_p_ is identical and is denoted with ϕ_p_ subsequently. H_p_ diffuses into the soil and induces eddy currents in the conductive subsurface, which generate a secondary magnetic field H_s_, shifted in phase by 90° with respect to H_p_ at the position of the Rx coil. The measured magnetic field (H_m_ = H_p_ + H_s_) at the Rx coil induces a voltage U_m_ with corresponding phase ϕ_m_. After amplification (AMP), U_m_ is fed together with U_p_ to the data acquisition (DAQ) unit. An ADC digitalizes both signals, and after further computation, the observed phase ϕ_o_, which is the difference ϕ_o_ = ϕ_m_ − ϕ_p_ between H_m_ and H_p_, is computed at the output, assuming that H_p_ and the reference signal I_p_ have the same phase ϕ_p_. It should be noted that each of the system components can cause parasitic phase drifts and phase offsets.

### 2.2. Phase Drift Model

**Figure 4:**
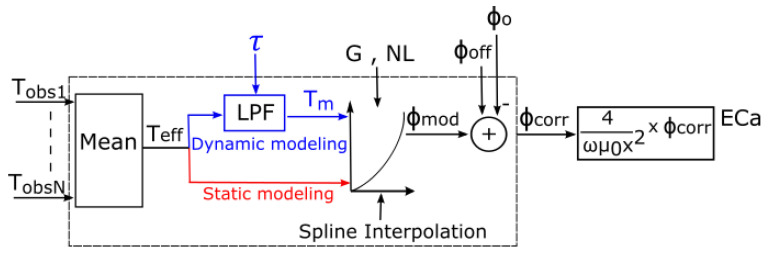
Schematic overview of the phase drift model. The mean T_eff_ of the selected observed temperatures T_obs1_ to T_obsN_ is used as input for the low-pass filter (LPF). The LPF output is a modeled temperature T_m_, which is converted into a phase ϕ_mod_ by spline interpolation with three reference points. ϕ_mod_ is subtracted from the observed phase ϕ_o_ and gives the corrected phase ϕ_corr_, which is converted to ECa by the McNeill [12] approximation. The system’s offsets are also considered by including a parameter ϕ_off_. The inclusion of the LPF results in dynamic modeling (blue path) and bypassing the LPF results in static modeling (red path), whereby phase calibration is done by the look-up table alone.

To predict the temperature drift characteristics of EMI measurements, we propose the phase drift model shown in Figure 4. The model is composed of two paths, a dynamic phase drift model (blue path) and a static phase drift model (red path), which only differ in either applying the low-pass filter (LPF) to the input or not. The LPF is used to determine the delayed response of the internal temperature of the system components to external temperature changes. To facilitate the interpretation of temperature information in terms of phase values, a look-up table with cubic spline interpolation is used. These two components (LPF and look-up table) make up the complete dynamic phase drift model and are discussed in more detail in the following section. For the static phase drift model, the LPF is bypassed, such that the measured temperatures are directly converted into modeled phase values using the look-up table. The phase drift model is controlled by the calibration parameters: the time constant τ of the LPF, the phase offset ϕ_off_ of the system, the gain G and a non-linear term NL.

#### 2.2.1. Effective Temperature Variation

The effective temperature variation (T_eff_) inside the EMI device can be determined by evaluating the mean value of various temperatures across the device. For this purpose, temperature sensors are selected which, on the one hand, have fast response times to the external temperature change and, on the other hand, are distributed over the entire length of the instrument so as to obtain a representative value for the internal temperature. Furthermore, the selection excludes sensors that show deviating temperatures due to local heating or cooling. The average value over the selected sensors provides the effective time series of the temperature for the model. In terms of statistical uncertainty, the mean value is also more accurate than the signals from the individual sensors.

By calculating the correlation coefficients of the selected temperature time series, it is checked whether the changes in the temperature values are uniform, i.e., that the selected sensors change identically with the external temperature and thus the condition for a representative temperature signal applies to the entire interior.

#### 2.2.2. Low-Pass Filter

To determine the influence of fast external temperature variations on the internal temperatures of the measurement system and components, a time domain digital LPF was used. The input and output signals of the LPF here are both temperature signals. In this paper, an infinite impulse response (IIR) filter is used, which filters an input signal using the present and delayed forms of the input signal in addition to a delayed form of the output signal. An IIR filter with a general time domain input and output relationship at time n is represented by the following difference equation (e.g., [27,28,29]):

(1) $$y\left(n\right)=\sum_{i=0}^{M}b_{i}x\left(n-i\right)-\sum_{i=1}^{N}a_{i}y\left(n-i\right)$$

where a_i_ and b_i_ are the filter coefficients, x(n) is the present and the past M input signals, and y(n) is the past N output signals. The Z-transformed input–output relationship of a dynamic system such as that given by Equation (1) in the time domain is:

(2) $$G\left(z\right)=\frac{Y\left(z\right)}{X\left(z\right)}=\frac{\sum_{i=0}^{M}b_{i}z^{-i}}{\sum_{i=0}^{N}a_{i}z^{-i}}=\frac{b_{0}+b_{1}z^{-1}+\ldots +b_{M}z^{-M}}{a_{0}+a_{1}z^{-1}+\ldots +a_{N}z^{-N}}$$

where z is the complex Z-transformed variable. To design a first-order digital IIR filter (M = 1, N = 1 in Equation (2)), it is customary to use an analog equivalent simple first-order LPF with a transfer function H_a_ given by:

(3) $$H_{a}\left(s\right)=\frac{\omega_{c}}{s+\omega_{c}}\;\;,\;\;\;\omega_{c}=\frac{1}{\tau }$$

where ω_c_ is the cut-off frequency of the LPF, the subscript a stands for analog, s is the Laplace operator, and τ is the time constant of the LPF. The digitization of the analog filter is achieved by bilinear transformation, which maintains the frequency characteristics of the filter. This is done by replacing the Laplace operator s by the term

(4) $$s=\frac{2}{T}\frac{z-1}{z+1}$$

where T is the sampling period. The Z-transform of the transfer function for the digital IIR filter is given as:

(5) $$H_{d}\left(z\right)=\frac{b_{0}+b_{1}z^{-1}}{1-a_{1}z^{-1}}$$

where the subscript d stands for digital. By substituting the value of s in Equation (4) into Equation (3) and comparing the results with Equation (5), the filter coefficients of the first-order digital filter can be determined:

(6) $$b_{0}=b_{1}=\frac{\frac{\omega_{c}T}{2}}{1+\frac{\omega_{c}T}{2}},{\;a}_{1}=\frac{1-\frac{\omega_{c}T}{2}}{1+\frac{\omega_{c}T}{2}}$$

where a_1_, b_0_ and b_1_ are the filter coefficients determined from the cut-off frequency ω_c_ and the sampling period of T. Using these filter coefficients, the following filter function can be derived from Equation (1) with modeled temperature T_m_:

(7) $$T_{m}\left(n\right)={\;b}_{0}T_{\mathrm{eff}}\left(n\right)+b_{1}T_{\mathrm{eff}}\left(\;n-1\right)+a_{1}T_{m}\left(n-1\right).$$

T_m_ is obtained at sampling point n using the effective input temperature T_eff_.

#### 2.2.3. Phase Value Calculation and Correction

Cubic spline interpolation with three reference temperature points ($T_{{\mathrm{ref}}_{\min}}=0.0$ °C, $T_{{\mathrm{ref}}_{\mathrm{mid}}}=25.0$ °C and $T_{{\mathrm{ref}}_{\max}}=50.0$ °C) with their corresponding reference phases $\phi_{{\mathrm{ref}}_{\min}}$, $\phi_{{\mathrm{ref}}_{\mathrm{mid}}}$ and $\phi_{{\mathrm{ref}}_{\max}}$, respectively, is used to convert the modeled temperature *T_m_* into a modeled phase for correction of the observed phase. This approach is also intended to account for the non-linear relationships between temperature and phase.

The reference temperature points span the observed ambient temperature range during EMI field measurements. The three corresponding phase points are calculated using ϕ_off_, G and NL. The first calibration phase point is the phase at 0.0 °C and represents the phase offset:

(8) $$\phi_{{\mathrm{ref}}_{\min}}=\phi_{\mathrm{off}}\;$$

The third calibration point is calculated from the gain while considering the phase offset:

(9) $$\phi_{{\mathrm{ref}}_{\max}}=\phi_{\mathrm{off}}+G\left(T_{{\mathrm{ref}}_{\max}}-T_{{\mathrm{ref}}_{\min}}\right).$$

The middle calibration point is determined from the non-linear term NL:

(10) $$\phi_{{\mathrm{ref}}_{\mathrm{mid}}}=\phi_{\mathrm{off}}+\mathrm{NLG}\left(T_{{\mathrm{ref}}_{\mathrm{mid}}}-T_{{\mathrm{ref}}_{\min}}\right)$$

**Figure 5:**
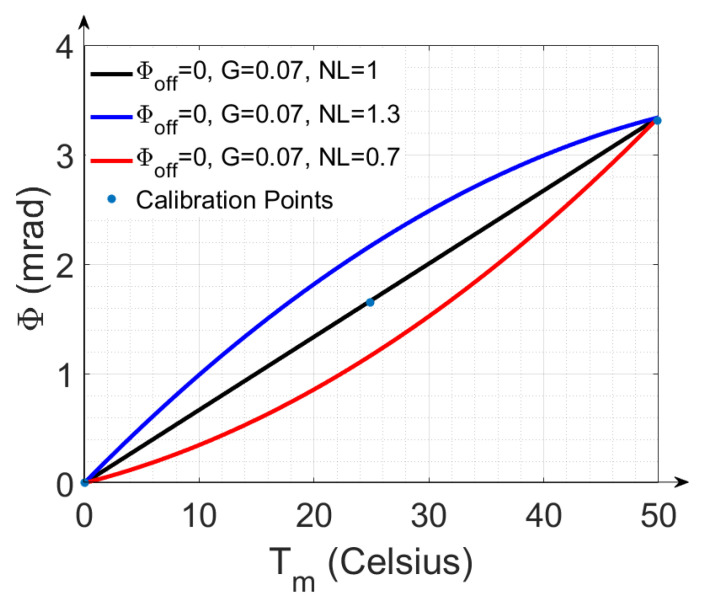
Graphical representation of cubic spline interpolation with three reference points over a temperature range of $T_{{\mathrm{ref}}_{\min}}=0\;$ °C to $T_{{\mathrm{ref}}_{\max}}=50.0\;$ °C. Three cases are displayed. When NL = 1, a linear relationship is obtained (black line). Any variation of NL away from 1 generates a non-linear relationship (blue and red line).

An NL value of 1 indicates a strictly linear behavior. Figure 5 shows three possible cases with the corresponding calibration points for the selected temperature range. Setting an NL value different from 1 will generate a non-linear temperature–phase relationship after spline interpolation, which will shift the curve either upwards or downwards.

Finally, to obtain the corrected phase

(11) $$\phi_{\mathrm{corr}}=\phi_{o}-\phi_{\mathrm{mod}}$$

The modeled phase ϕ_mod_ is subtracted from the measured phase ϕ_o_.

### 2.3. ECa Calculation

The apparent electrical conductivity (ECa) is obtained using the approximation proposed by McNeill [12], where the imaginary component of the magnetic field sensed by the EMI instrument varies linearly with ECa [12]:

(12) ECa=4ωμ0x2Im(HsHp)

where ω is the angular frequency, μ_0_ is the conductivity of free space, and x is the intercoil spacing. For a small ratio of Im(H_s_/H_p_), the ratio corresponds to the phase ϕ between H_s_ and H_p_ (Im(H_s_/H_p_) = tan(ϕ) ≈ ϕ). Based on the system description from Figure 3, the ratio Im(H_s_/H_p_) is equal to Im((H_s_ + H_p_)/H_p_) and therefore equal to the phase difference ϕ between the measured phase ϕ_m_ of the magnetic field (H_p_ + H_s_) and the phase ϕ_p_ of the primary field H_p_. Equation (12) can therefore be simplified by the small phase angle approximation:

(13) $$\mathrm{ECa}=\frac{4}{{\omega \mu }_{0}x^{2}}\phi$$

This method for ECa calculation relies on the low induction number approximation (LIN). The LIN approximation is applicable to measurements performed on or above the ground surface with small intercoil spacings between Tx and Rx coils, low soil conductivities and at low frequencies [12]. Equation (13) is now used to estimate the necessary phase measurement accuracy. The approximated ϕ increases monotonically to the square of the separation between the Tx and Rx coils. With respect to the system in Figure 2, very small phase values in the order of ~28 μrads are obtained for an intercoil spacing of 1.2 m and an ECa of 1 mSm^−1^. Such accuracy of 28 μrads can only be achieved with additional drift correction.

### 2.4. Drift Calibration Measurements

Calibration measurements are necessary to determine the calibration parameters of the phase drift model. For this purpose, the influence of the soil on the system’s thermal drift should be as small as possible and the calibration method should be practicable in the field. Hence, the EMI instrument was mounted on a wooden rack and elevated to a height of 0.70 m in a garden near Niederzier, Germany in July 2020. Care was taken to ensure that there were no power lines in the vicinity of the instrument to avoid interferences. To investigate the temperature effects on the measurement system, sixteen sets of ECa measurements were performed in VCP configuration for 30 h (i.e., 16 day and night cycles). The measured temperature inside the EMI instrument ranged from approximately 25 °C to 58 °C during the measurements. The height of 0.70 m was used to provide a suitable compromise between ease of experimentation and the minimization of soil effects on the observed data. The calibration measurements were preferably done in the VCP configuration because changes in soil properties (e.g., temperature and water content) have the lowest effect on observed ECa for the given height [23]. After the measurements, the model parameters were determined for each of the 16 datasets.

Appropriate values for the calibration parameters m = (ϕ_off_, τ, G, NL) of the phase drift model were estimated by minimizing the misfit between observed phase ϕ_o_ and modeled phase ϕ_mod_ using an objective function with the L2-norm:

(14) $$L_{2}\left(m\right)={\left|\left|\phi_{o}-\phi_{\mathrm{mod}}\left(m\right)\right|\right|}_{2}={\left|\left|\phi_{\mathrm{corr}}\left(m\right)\right|\right|}_{2}.$$

The Simplex algorithm [30] was used to minimize this objective function. The starting values for the minimization were calculated by fitting a linear model to the phase and temperature data, which provided initial values for ϕ_off_ and G. The time constant τ (in seconds) and non-linear term NL were both assigned an initial value of one. For further evaluation of the optimization, the root mean square error RMSE = $\sqrt{\frac{\sum_{1}^{n}\left(\phi \mathrm{corr}\left(n\right)\;–\;\mathrm{mean}\left(\phi \mathrm{corr}\left(n\right)\right)\right)2}{n}}$ is considered.

## 3. Results and Discussion

### 3.1. Measured Temperature Distribution

**Figure 6:**
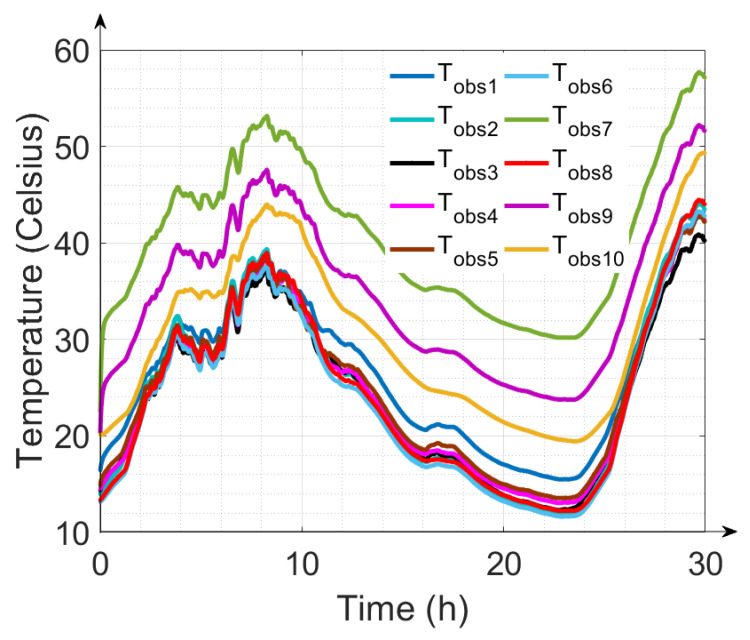
Time series of observed temperature T_obs_ obtained for calibration measurement #16. The 10 temperature sensors were spread across the EMI instrument and measured temperature variation in the instrument.

The observed temperature for all ten sensors (T_obs_) for calibration measurement #16 is exemplarily shown in Figure 6. The temperatures shown are almost the same in terms of variation. However, sensors 7, 9 and 10 have higher overall temperature values than the other sensors due to local self-heating of the modules to which the sensors are attached. These sensors do not represent the uniform internal temperature of the device and are therefore not considered for drift correction. In addition, the temperature curves of sensors 1 and 3 indicate delayed reaction times. These sensors were therefore also excluded from the analysis.

Finally, sensors 2, 4, 5, 6, 8 were used to calculate the effective time series for the model for all measurements. To check whether all selected sensors always showed the same temperature variations, i.e., whether a uniform temperature variation was measured in the entire device, the correlation coefficients of the selected temperatures were calculated. The smallest correlation coefficient between the selected temperature curves of all 16 measurements was never less than 0.994, indicating a uniform temperature inside the system.

### 3.2. Performance of Calibration

The 16 calibration measurements were analyzed separately to evaluate the robustness of the calibration method. Table 1 shows the results of fitting each individual calibration measurement, as well as the respective root mean square error (RMSE1) of the ECa values. The RMSE1 varied from 0.31 mSm^−1^ to 0.56 mSm^−1^ across all 16 datasets, with a median of 0.42 mSm^−1^ (equivalent phase of ~11.9 μrad). The calibration parameters τ, G and NL were similar for all calibration measurements, which indicates the reliability of the calibration method and calibration approach. The time constant τ is an indication of the response time of the individual components of the measurement system to temperature changes. The median τ suggests that it takes ~1107 s (~18 min) for the components of the measurement system to respond to the internal measured temperature. It is expected that this delay is mostly associated with the coils due to their large thermal inertia [13,15]. The fitted G is expressed as a drift in ECa, and it varied around a median value of 2.27 mSm^−1^K^−1^ (phase of ~64.5 μrad/K). Accurate determination of G is extremely important for the drift correction. The standard deviation (std) of the fitted ECa drift was only 0.03 mSm^−1^K^−1^, which is low relative to the median *G*. NL varied around a median value of 1.19, which means that the phase values are non-linearly related to temperature (Figure 5), showing that this non-linear term is also necessary for the correction. By setting NL to 1.0 (i.e., a linear model), the median of the RMSE1 increased from 0.42 mSm^−1^ to 1.2 mSm^−1^.

The median values of the calibration parameters obtained from the 16 datasets were also applied to the 16 datasets, which resulted in 16 additional root mean square error (RMSE2) values that were slightly higher than the RMSE1 values. The median value of these RMSE2 values obtained using a single set of calibration parameters was 0.48 mSm^−1^, which is only slightly higher than the median value of the RMSE1 values obtained using the individual calibration parameters, which was 0.42 mSm^−1^. The similar results obtained for both individual and median calibration parameters over 16 days show the reproducibility of the calibration parameters and thus also the long-term calibration ability of the system.

**Figure 7:**
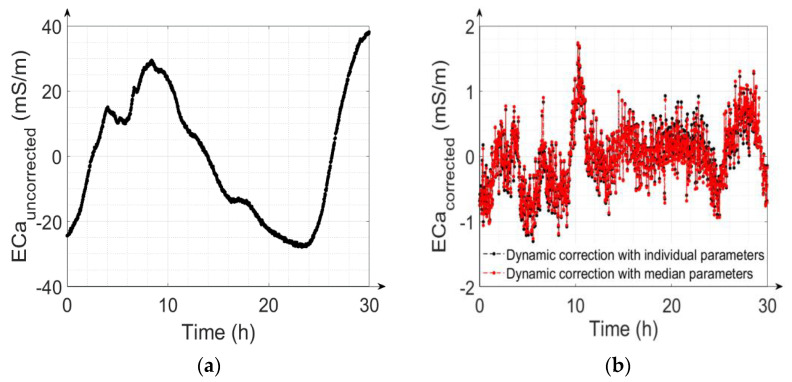
(**a**) Uncorrected ECa for calibration measurement #16; (**b**) corrected ECa for measurement #16 using individually calibrated parameters and using the median of all calibrated parameters. The ECa values were shifted to have a zero mean to focus on the changes in ECa instead of the absolute values.

The calibration ability and the efficiency of the drift correction can also be demonstrated using the time series of the ECa values. For instance, the uncorrected and corrected ECa values of measurement #16 using the individual calibration parameters, as well as the median calibration parameters, are shown in Figure 7a,b, respectively. The peak-to-peak ECa value of the uncorrected data in Figure 7a is approximately 62 mSm^−1^, while the ECa value of the corrected data is approximately 3 mSm^−1^. Figure 7b shows furthermore that the ECa values for the individual and median calibration parameters are similar. The RMSE of the corrected ECa values is 0.48 mSm^−1^ for individually calibrated parameters and 0.49 mSm^−1^ for the median calibration parameters.

### 3.3. Advantage of Implementing the LPF in the Drift Correction Model

**Figure 8:**
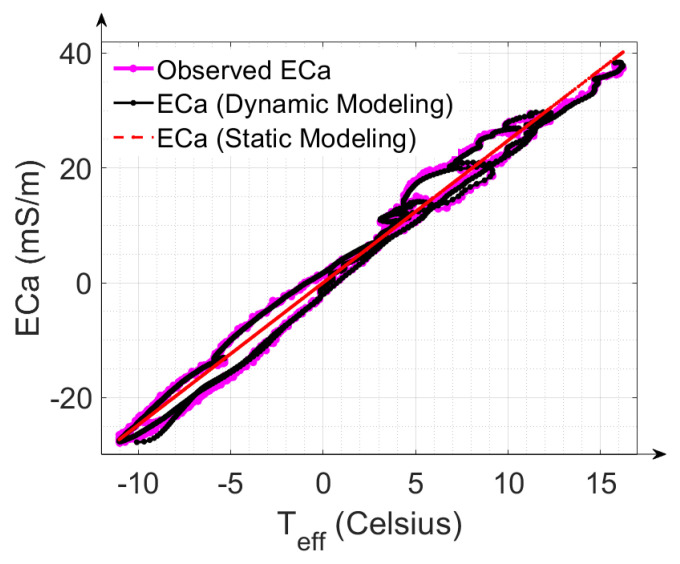
Observed ECa values (pink plot), ECa values generated from dynamic modeling (black plot) and ECa values generated from static modeling (red plot) represented as a function of effective temperature T_eff_ for calibration measurement #16. The ECa values were shifted to have a zero mean to focus on the changes in ECa instead of the absolute values.

In this section, we will demonstrate the advantages of dynamic correction with an LPF in comparison to the static correction of temperature drift without an LPF. Figure 8 shows the observed ECa, the ECa obtained from dynamic correction and the ECa obtained from static correction (all shifted to have a mean of zero to represent ECa differences instead of absolute values) as a function of temperature. The occurrence of temperature-dependent hysteresis loops arising from the fluctuation of the internal ambient temperature of the measurement device is apparent. The hysteresis loops are an accumulation of present and prior warming and cooling cycles, as also reported by Huang et al. [22]. Figure 8 shows that the static approach alone is not sufficient to effectively analyze the measurement device’s drift properties over time, as it does not reproduce the hysteresis loops present in the measured data. Applying the dynamic approach enables the reproduction and correction of these hysteresis effects.

**Figure 9:**
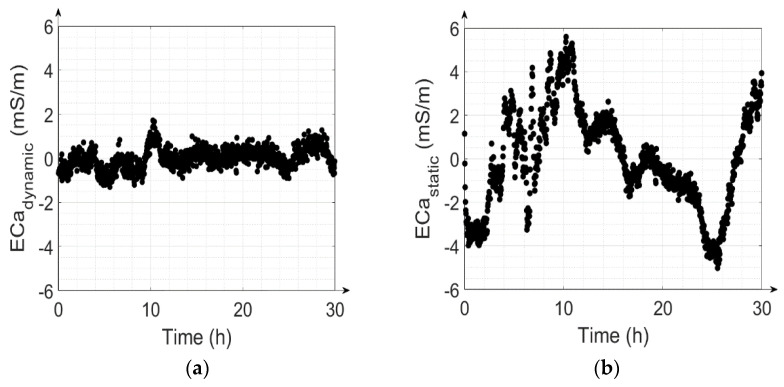
Individually corrected ECa values calculated using (**a**) the LPF (dynamic drift correction); (**b**) no LPF (static drift correction). The ECa values were shifted to have a zero mean to focus on the changes in ECa instead of the absolute values.

To illustrate the importance of the LPF, the effects of correction with the dynamic (Figure 9a) and static approach (Figure 9b) on the measured time series of measurement #16 are shown and the RMSE of the two cases for single parameter correction are also analyzed. The peak-to-peak range of ECa values obtained with static correction is around four times higher than the range obtained with dynamic correction, and the RMSE1 increases to 1.94 mSm^−1^ when the LPF is not taken into account, compared to 0.42 mSm^−1^ with dynamic correction.

### 3.4. Effect of Soil Conductivity Changes on the Calibration

To investigate the possible influence of temperature-related changes in soil properties on the presented instrument calibration, a two-layer model was assumed, where the first layer with a thickness of 0.70 m consisted of air (0 mSm^−1^) and the second layer with infinite thickness consisted of a homogeneous soil. The sensitivity of the measurement system was modeled using the cumulative response function (CRF) described by McNeill [12]. This analysis showed that an increase in soil electrical conductivity (in the first few meters) by 1 mSm^−1^ resulted in an increase in measured ECa of 0.37 mSm^−1^ for the VCP configuration with an intercoil spacing of 1.20 m at a height of 0.70 m. If it is now considered that the soil conductivity changes by around 2% per K [1], then the expected ECa drift due to the effect of temperature on soil conductivity is 0.74% per K. This value should be considered as a worst-case scenario since the soil will not be heated instantaneously. By assuming a typical soil electrical conductivity (EC) of 10 mSm^−1^ and considering that the relative ECa change due to soil temperature is less than 0.74 % per K of the soil EC, the expected worst-case ECa change due to soil temperature change is ~0.07 mSm^−1^K^−1^. Compared with the system drift of 2.27 mSm^−1^K^−1^, it is safe to conclude that for an intercoil spacing of 1.2 m, the effect of changes in the soil electrical conductivity during the calibration measurements is very small and can therefore be neglected. For smaller coil spacings, the influence of the soil becomes even smaller. For example, at a smaller intercoil spacing of 0.4 m, the expected ECa change due to soil temperature change is only ~0.03 mSm^−1^K^−1^ and the expected system drift is ~20.4 mSm^−1^K^−1^, assuming that the measured phase values of the system drifts are independent of intercoil spacing. For larger intercoil spacings, the effect of the soil becomes stronger because of the increased sensing depth. For instance, at an intercoil spacing of 6 m, the expected ECa change due to soil temperature change would be ~0.15 mSm^−1^K^−1^ and the expected system drift is only 0.09 mSm^−1^K^−1^. The system drift for such large coil spacing is even smaller as there is an inverse square relationship between the ECa values and phase values (Equation (13)). Note that these estimates are all worst-case estimates because it is assumed that soil temperature changes uniformly throughout the entire sensing volume. In reality, only the temperature of the top soil will change.

## 4. Conclusions

A novel temperature-dependent drift correction method for electromagnetic induction (EMI) measurements that accounts for the transient response of system components to varying ambient temperature conditions was introduced. The method makes use of a low-pass filter (LPF) to reproduce the dynamic characteristics of measured drifts. To verify the correction method, a customized EMI instrument at a height of 0.70 m above ground was used to simultaneously measure the apparent electrical conductivity (ECa) and internal ambient temperature across the device using sensors at ten locations. The measurement system used is optimized for low drift, but does not use internal drift correction circuitry. In total, 16 measurements with a respective duration of 30 h were measured and evaluated, showing a median drift of approximately 2.27 mSm^−1^K^−1^ with a standard deviation (std) of 30 μSm^−1^K^−1^ between different measurements. Without further corrections, drifts of 2.27 mSm^−1^K^−1^ would falsify the measurements extremely at varying outdoor temperatures. For this reason, commercial EMI systems typically use further internal correction circuits or additional correction tables. However, the results presented here show that these drifts are quite stable, with a variation of only 30 μSm^−1^K^−1^, and can be corrected for the most part with the shown correction method. After correcting the 16 calibration datasets with the median value of all calibration parameters, the median RMSE of all data sets is only 0.48 mSm^−1^. When compared with raw observed data, the dynamic approach corrects observed ECa values by a factor of 30. The results also showed a delayed response of the dominant drift source of around 18 min to the internal temperature changes. The relevance of considering this delay was shown by comparing results obtained with static modeling and correction, which resulted in an RMSE with a median value of 1.94 mSm^−1^. The presented results show that the novel model-based dynamic drift correction method using LPF offers four times higher accuracy in drift correction compared to static correction and is a reliable approach for mitigating temperature drift effects in EMI data, even in the case of the rapid temperature changes that may occur in typical field measurements. In addition, the model-based calibration method presented here no longer requires stable temperature levels, as is the case with typical calibration in thermostatically controlled rooms. It can therefore be applied very easily, without much effort. Simple outdoor measurements with a sufficient temperature response are perfectly sufficient for calibration. With the assistance of manufacturers, the novel correction method could be easily applicable to state-of-the-art commercial EMI systems. With the required temperature sensors installed on the instruments and the application of the novel dynamic correction method, the accuracy of temperature-related drift correction in EMI systems can be increased in comparison to standard static correction. This correction method could be useful for a wide range of agricultural applications where near-surface ECa measurements are needed.

## Figures and Tables

**Table 1:** Calibration parameters time constant (τ), Gain (G converted to ECa) and non-linear term (NL) and the resulting root mean square error (RMSE) for all 16 calibration measurements. The median and standard deviation (std) of the fitted parameters are also provided.

Data	τ (s)	G (mSm^−1^K^−1^)	NL	RMSE1 (mSm^−1^K^−1^)	RMSE2 (mSm^−1^K^−1^)
1	1201.10	2.27	1.17	0.36	0.37
2	1176.82	2.36	1.05	0.39	0.42
3	1038.07	2.25	1.18	0.40	0.47
4	968.33	2.23	1.22	0.40	0.64
5	1198.90	2.25	1.18	0.41	0.46
6	1076.17	2.25	1.19	0.31	0.32
7	1121.04	2.25	1.08	0.31	0.42
8	1038.55	2.24	1.24	0.44	0.59
9	1147.57	2.28	1.25	0.39	0.48
10	1154.75	2.26	1.16	0.56	0.61
11	1122.22	2.26	1.21	0.39	0.39
12	1041.29	2.26	1.22	0.37	0.47
13	1152.85	2.25	1.18	0.55	0.57
14	1007.87	2.29	1.25	0.37	0.41
15	1177.05	2.32	1.29	0.53	0.63
16	1104.52	2.26	1.20	0.48	0.49
median	1107.94	2.27	1.19		
std	71.78	0.03	0.06		

## Data Availability

The authors confirm the availability of the data supporting the reported findings in this study. Raw data were acquired through measurements performed at the Central Institute of Engineering, Electronics and Analytics (ZEA-2) and are available upon request.
